# Neonatal Infectious Disease: A Major Contributor to Infant Mortality Requiring Advances in Point-of-Care Diagnosis

**DOI:** 10.3390/antibiotics13090877

**Published:** 2024-09-13

**Authors:** Mary Garvey

**Affiliations:** 1 Department of Life Science, Atlantic Technological University, F91 YW50 Sligo, Ireland; mary.garvey@atu.ie; Tel.: +71-9305529; 2 Centre for Precision Engineering, Materials and Manufacturing Research (PEM), Atlantic Technological University, F91 YW50 Sligo, Ireland

**Keywords:** neonate, mortality, infection, resistance, point of care

## Abstract

Neonatal infectious disease continues to result in high rates of infant morbidity and mortality. Early- and late-onset disease represent difficult to detect and difficult to treat illnesses, particularly when antimicrobial resistant pathogens are present. Newborns are immunodeficient and are at increased risk of vertical and horizontal infection, with preterm infants increasingly susceptible. Additional risk factors associated with infection include prolonged use of a central catheter and/or ventilation, congenital abnormalities, admittance to intensive care units, and the use of broad-spectrum antibiotics. There is increasing recognition of the importance of the host microbiome and dysbiosis on neonatal infectious disease, including necrotising enterocolitis and sepsis in patients. Current diagnostic methods rely on blood culture, which is unreliable, time consuming, and can result in false negatives. There is a lack of accurate and reliable diagnostic tools available for the early detection of infectious disease in infants; therefore, efficient triage and treatment remains challenging. The application of biomarkers, machine learning, artificial intelligence, biosensors, and microfluidics technology, may offer improved diagnostic methodologies. Point-of-care devices, such diagnostic methodologies, may provide fast, reliable, and accurate diagnostic aids for neonatal patients. This review will discuss neonatal infectious disease as impacted by antimicrobial resistance and will highlight novel point-of-care diagnostic options.

## 1. Introduction 

Infectious disease in infants is a significant cause of morbidity and mortality in the first 28 days of life (neonatal period), accounting for 33% of neonatal deaths and 50% of deaths in children under 5 [1]. Furthermore, patients who survive often manifest with several neurodevelopmental difficulties and chronic disabilities if detection and treatment does not occur rapidly [2]. Neonatal infection is either early-onset resultant from vertical transmission, such as transplacental spread late in pregnancy, ascending infection during labour and delivery, or late-onset nosocomial transmission, occurring horizontally at the postnatal stage [3]. Eliminating viral pathogens in the etiology of disease is also important in disease triage. The World Health Organisation (WHO), however, states that neonatal infectious are primarily bacterial in origin, manifesting as pneumonia, sepsis, and meningitis, causing over 550,000 neonatal deaths annually [4]. Neonatal sepsis is the most serious manifestation, with a mortality rate of 50% and an incidence of ca. 1.2% in developed countries and ca. 40% in developing and low-to-middle income countries (LMIC) [5]. Sepsis is defined as a dysregulated host response to systemic infections leading to shock and multiorgan dysfunction, failure, and often death [6]. Gram-positive bacteria (GPB) associated with infection include *Streptococcus agalactiae*, *Listeria monocytogenes*, *Staphylococcus aureus*, and methicillin resistance *S. aures* (MRSA) [7]. Gram-negative bacteria (GNB) associated with infection include members of the Enterobacteriaceae family, namely *Escherichia coli*, *Klebsiella* spp., *Enterobacter* spp., and *Serratia* spp., and other species, including *Pseudomonas aeruginosa* and *Acinetobacter baumannii* [5]. Importantly, hypervirulent strains of *K. pneumoniae* have emerged, which possess additional virulence factors that increase pathogenicity, with resistance to cephalosporin, cephamycin, monobactam, aminoglycosides, fluoroquinolone, and trimethoprim–sulfamethoxazole [6]. *Neisseria gonorrhoea* is also associated with newborns, causing neonatal septic conjunctivitis in particular [8] (Table 1). Congenital syphilis (CS) is caused by the vertical transmission of Gram-negative *Treponema pallidum*, resulting in a multiorgan infection, neurologic and musculoskeletal disabilities, and neonatal fatalities [9]. CS is believed to result in ca. 370,000 perinatal deaths and 21,500 further CS-related deaths within 1 year post-birth, associated with prematurity, a low birth weight, and congenital infections [9]. Fungal infectious disease also represents a serious risk and challenge in neonates, in terms of detection, treatment, and mortality. For example, *Candida* species are associated with 93% of fungal bloodstream infections (BSIs) and late-onset sepsis in neonates, with mortality rates of ca. 70% [10]. *Aspergillus* fungi are the most frequent causes of neonatal invasive fungal infections (IFIs) in premature infants, with high rates of mortality [11]. The risk factors associated with neonatal infectious disease include premature birth, congenital abnormalities, admittance to intensive care units (ICUs), and invasive care with the use of medical devices, including central venous catheters (CVCs) and respiratory support devices [7]. Pathogen-specific virulence factors, coupled with antimicrobial resistance (AMR), also contribute to infectious disease morbidity and prognosis. There is also increasing recognition of the importance of the host microbiome and dysbiosis on neonatal infectious disease. Intestinal dysbiosis in neonates has been associated with necrotising enterocolitis (NEC) and sepsis in patients [12]. The causative pathogens associated with ICU infections include *S. aureus*, MRSA, *Klebsiella* species, *E. coli*, *Pseudomonas* species, *Acinetobacter*, and *Enterococcus* species [13] (Table 1). 

The guidelines entitled ‘Neonatal infection: antibiotics for prevention and treatment’, published by the National Institute for Health and Care Excellence (NICE), which were updated in March 2024, outline infection mitigation protocols for neonates and expectant mothers, to improve timely disease recognition and reduce antibiotic use in infant care [14]. Early detection and pathogen identification are key to the successful treatment of disease and are achieved via sampling of blood, cerebrospinal fluid (CSF), and bronchoalveolar lavage fluid (BALF) [2]. A significant number of pathogens, however, ca. 60%, cannot be detected through these mediums, with negative blood cultures a common occurrence with higher mortality rates [15]. Currently, antibiotic treatment of disease is the mainstay of infection management in neonates, which is impacted by AMR. The WHO recommends providing prophylactic intramuscular (IM) or intravenous (IV) ampicillin and gentamicin in neonates who display risk factors of neonatal infection for 48 h, which should be continued with symptoms of sepsis or positive blood culture [16]. For neonates with serious bacterial infection, hospitalisation and IM or IV antibiotic therapy (gentamicin and benzylpenicillin or ampicillin) for 7–10 days is recommended [17]. Sustainable Development Goal (SDG) number 3 on good health and wellbeing aims to “end preventable deaths of newborns and children under 5 years of age, with all countries aiming to reduce neonatal mortality to at least as low as 12 per 1000 live births and under-5 mortality to at least as low as 25 per 1000 live births” by 2030 [18]. The SDGs are targets for global development that were adopted in 2015 and should be achieved by all countries by 2030. They “are a call to action to end poverty and inequality, protect the planet, and ensure that all people enjoy health, justice and prosperity” [18]. 

Advancements in disease surveillance methods, such as sensitive and accurate biomarkers, proteomics, and machine learning (ML), have demonstrated the potential to improve neonatal infection detection. The speedy detection and diagnosis of infectious disease in neonates is essential to inform clinical treatment and to safeguard infant life in line with SDG 3. This timely review aims to discuss neonatal infectious disease prevalence, aetiology, and recent advances in terms of disease diagnosis and point-of-care protocols.

**Table 1:** Pathogens associated with neonatal infectious disease, mortality, and evident AMR profiles.

Category	Gram Stain	Species	Mortality	AMR Emerging and Present
Bacterial	Gram negative	*P. aeruginosa*	56% [19]	Colistin-resistant *P. aeruginosa*, pan-drug-resistant strain [20,21]
		*E. coli*	>35% within 30 days for BSIs, 14–25% for meningitis [22]	Aminoglycosides, cephalosporins, penicillin, fluoroquinolones, B-lactam combination agents [21], plasmid mediated colistin resistant [23]
		*K. pneumoniae*	13% BSI/sepsis [19]	Cephalosporins, fluoroquinolones, trimethoprim/sulfamethoxazole, colistin [20]; extended spectrum beta lactamase (ESBL) detected conferring resistance to penicillins, cephalosporins, including third-generation cephalosporins, plasmid mediated colistin resistant [23]
		*A. baumannii*	30-day mortality rate 36.3% [24]	Carbapenem-resistant strains that produce metallo-beta-lactamase (MBLs), colistin [24]
		*N. gonorrhoea*	Not determined	Sulphonamides, penicillins, tetracyclines, macrolides, and fluoroquinolones [8]
		*T. pallidum*	20% [9] in <28-day-old neonates	Macrolide and tetracycline resistance [25]
	Gram positive	*S. pyogenes*	5–20% and ca. 45% for sepsis [26]	Strains isolated showing resistance to penicillin [26]
		*Enterococcus species—E. faecalis*	20% in septicaemia cases [27]	Vancomycin, beta-lactams such as cephalosporins, aminoglycosides, trimethoprim–sulfamethoxazole, and clindamycin
		*S. agalactiae* (group B *Streptococcus*)	8–13% for meningitis [22]	Emerging resistance to erythromycin, clindamycin, and fluoroquinolones [28]
		*S. aureus*	12% [19]	MRSA, Vancomycin resistant *S. aureus* (VRSA), [13]
		*L. monocytogenes*	10–50% early-onset infection [29]	Cephalosporin, fosfomycin, and fluoroquinolones, gentamicin combined with ampicillin/amoxicillin does not affect *Listeria* within macrophages [29]
Fungal		*C. auris*	Sepsis 70% [10]	Resistance to azole [30], echinocandins, pyrimidines, and polyenes common in clinical isolates [31]
		*C. albicans*	70% BSI, sepsis [30]	
		*C glabrata*		
		*C. tropicalis*	28.7% [30]	
		*A. fumigatus*	ca. 70% IFIs	Emergence of Azole resistance
		*C. neoformans*	66% in mothers with HIV [32]	Caspofungin [31]

## 2. Neonatal Infectious Disease

Neonatal infections are categorised as early onset, developing within the first 72 h of life [14], and the infection is vertical, or late-onset infections, developing after 72 h, and the infection is horizontal. Horizontal transmission occurs from healthcare staff and fomites and medical devices acting as reservoirs of pathogenic species. Newborns are more susceptible to infectious disease due to their reduced immune capacity and exposure to nosocomial pathogens, with patients in ICUs also exposed to the systematic and chronic use of invasive medical devices (e.g., central lines), prolonged hospitalisation, and co-morbidities [19]. Low and very low birth weight (VLBW) and preterm infants (infants born before 37 weeks of pregnancy) admitted to neonatal ICUs are particularly vulnerable. The innate components of immunity, namely the skin and mucous membranes, of premature neonates have increased pathogen permeability, an insufficient reserve of preformed neutrophils, immature granulocyte migration and bacterial phagocytosis, and decreased immunoglobulins, limiting their immune response to infection [33].

Additionally, the increasing prevalence of AMR species and the emergence of multidrug resistant (MDR), extensive drug resistant (XDR), and pan-drug resistant pathogens, including MRSA, vancomycin resistant *S. aureus* (VRSA), extended spectrum beta lactamase (ESBL) producing *Enterobacteriaceae* and *Acinetobacter* species [13], and fungal *Candida* and *Aspergillus* species, has seriously impacted neonatal morbidity and mortality [21]. Reports show that ca. 214,000 neonatal deaths from sepsis are caused by AMR annually, with AMR in neonatal sepsis becoming more prevalent [34].

### 2.1. Bacterial Pathogens Associated with Neonatal Infectious Disease

Studies show that *S. agalactiae* and *E. coli* are the most frequently isolated pathogens in early-onset neonatal infections with *S. aureus*, coagulase negative *Staphylococci* (CoNS), *Enterobacteria*, and *Streptococcus pyogenes* associated with late-onset infections [7]. *S. pyogenes* is a beta-hemolytic bacteria known as serogroup A *Streptococcus*, which produce pyrogenic exotoxins, enabling tissue invasion and septic shock in patients [26]. With additional studies identifying *K. pneumoniae* and *E. faecalis* in both early- and late-onset cases [35]. CoNS are commonly associated with bloodstream infections (BSIs), with rates of 78% and 36% in developing and developed countries, respectively [19]. BSIs and medical device associated with BSIs, e.g., central line associated BSIs, are frequently causative of neonatal sepsis. In neonatal sepsis, GPB are more prevalent than GNB; GNB, however, are associated with increased mortality rates [34]. *Klebsiella* species, *S. aureus*, and *CoNS* are the most common pathogens of neonatal sepsis in developing countries [36]. The lipopolysaccharide (LPS) toxin, excreted by the cell membrane of GNB, is an important contributor to mortality in neonatal sepsis. Furthermore, the LPS toxin of *E. coli* has a greater immunogenic potential than other Gram-negative species, allowing for increased pathogenicity [21]. Gram-negative *P. aeruginosa* has a high mortality rate of ca. 56%, due to its potent virulence factors and MDR profile [19] (Table 2). Sepsis resulting from *A. baumannii* is associated with mechanical ventilation, neutropenia, thrombocytopenia, kidney injury in neonates, and incorrect antibiotic use, with carbapenem-resistant strains present [24]. Blood culture data of neonatal ICU patients have revealed that *S. aureus* and *Klebsiella* species were two of the most common causes of neonatal sepsis [13]. Studies have described a disease state manifesting with systemic exanthema, fever, low-positive serum C-reactive protein (CRP) values, and thrombocytopenia, within the first week of life as associated with MRSA selectively producing toxic shock syndrome (TSS) toxin-1 (TSST-1), which has superantigenic activity [37]. Group B *Streptococcus* (GBS) or *S. agalactiae* are common pathogens associated with early-onset neonatal meningitis, being responsible for >40% of all early-onset cases [38]. This is followed by *E. coli*, causing ca. 30% of early-onset meningitis in VLBW and preterm newborns [38]. *L. monocytogenes*, *Enterococcus* sp., and *S. pneumoniae* are also associated with some cases of early-onset neonatal bacterial meningitis. CoNS, *S. aureus*, *E coli*, and *K. pneumoniae* are associated with late-onset meningitis. Vertical transmission of *L. monocytogenes* is rare, but is associated with stillbirths, uterine foetal loss, abortion, neurodevelopmental issues, and neonatal mortality of 10 to 50% in early-onset disease [39]. The mortality from neonatal meningitis is significant at ca.15% in developed countries, with neurological issues developing in 20 to 50% of survivors, and ca. 58% in LMIC, with moderate-to-severe neurodevelopmental issues in ca. 23% of survivors [40]. *S. pneumoniae*, *S. pyogenes*, and *S. aureus* are associated with late-onset neonatal pneumonia. 

Additional species associated with ventilator-associated pneumonia (VAP) include *P. aeruginosa*, *Enterobacter* spp., and *Klebsiella* and *Enterococcus* spp., with 25% of VAPs being polymicrobial [33]. VAP in ICU patients has increased mortality compared to non-VAP cases [33], where biofilm formation on indwelling medical devices is associated with pathogenicity [20]. Neonatal pneumonia has a mortality rate of ca. 20% in developed countries and ca. 30% in developing countries [41]. Vertical transmission of *N. gonorrhoeae* is associated with neonatal fatalities, premature rupture of membranes, VLBW infants, preterm births, and neonatal septic conjunctivitis [8]. Similarly, the vertical transmission of *T. pallidum* or Syphilis during pregnancy can lead to stillbirth, miscarriage, and maternal and neonatal morbidity [42]. Vertical transmission of *Chlamydia trachomatis* is associated with pneumonia and neonatal conjunctivitis; the ease of treatment and screening in maternal patients, however, has a positive impact on neonatal patient prognosis [43]. The increase in MDR bacterial species, particularly GNB, is of great concern in neonatal patients, with the WHO listing carbapenem-resistant *A. baumannii* and *P. aeruginosa*, and carbapenem-resistant and third-generation cephalosporin-resistant Enterobacteriaceae as the most common emerging species [23].

**Table 2:** Outlining important virulence factors present in bacterial and fungal pathogens associated with neonatal morbidity.

Virulence Factor	Pathogen	Mode of Action
Capsular polysaccharide	*K. pneumoniae*, *hypervirulent K. pneumoniae*, group B Streptococcus, *Cryptococcus neoformans* [44]	Protects pathogen from opsonization and phagocytosis by macrophages and neutrophils and suppresses the early inflammatory response through the inhibition of IL-8 expression [45]
Fimbrial adhesins	*K. pneumoniae*, *E. coli*, CoNS, *Streptococcus* species [46]	Mediating the adhesion of the pathogen to the mucosal layer and/or epithelial cells [6]
Siderophores [45]	GPB and GNP, e.g., *K. pneumoniae* and most fungal species, e.g., *A. fumigatus*	Acquisition of iron used for reproduction
Biofilm formation	All relevant neonatal pathogenic bacterial and fungal species	Colonisation of biotic and abiotic surfaces including medical devices, increased resistance to therapeutics, biocides, and host immunity
Plasmids		Carries resistance genes in pathogenic species, which can be transmitted, e.g., carbapenem-resistant genes in *K. pneumonia*
Intracellular colonisation of macrophages	*L. monocytogenes*, *Chlamydia*, *C. albicans*, *C. neoformans* [44]	Immune avoidance [29]
Toxin production	Candidalysin produced by *C. albicans* [44], *Aspergillus* and *Fusarium* species produce mycotoxins, Lipopolysaccharide (LPS) produced by GNB, selective toxic shock syndrome (TSS) toxin-1 (TSST-1) produced by *S. aureus*, pyrogenic exotoxins produced by group A Streptococcus [26]	Permeabilization and invasion of epithelial cells [44]; nephrotoxic, genotoxic, teratogenic, carcinogenic, and cytotoxic [21]; binds with cell-mediated immune components, e.g., CD 14, CD 16, CD 18, humoral-mediated antibodies and lactoferrin, and activates Toll-like receptors (TLRs) inducing sepsis [21]; systemic exanthema, fever, low-positive serum C-reactive protein (CRP) values, and thrombocytopenia [37]; tissue invasion, septic shock

### 2.2. Fungal Pathogens Associated with Neonatal Infectious Disease

Fungal infections termed mycosis result in high rates of neonatal fatalities from fungal-induced blood stream infections, sepsis, and invasive fungal infections (IFIs). IFIs have a mortality of ca. 60% in ICU patients, with species such as *Candida* spp., *Aspergillus* spp., and *Cryptococcus* spp. involved. The mortality rate of Candidiasis is ca. 37% in developed countries and up to 75% in LMICs [47]. *C. albicans* and *C. glabrata* are the most common isolated *Candida* in BSIs and late-onset sepsis in neonates, with mortality rates of ca. 70% [21]. The MDR *C. auris* has emerged as a significant threat to neonates, resulting in systemic difficulties in treating infections [48]. Invasive Candidiasis is the third most common pathogen associated with late-onset sepsis in VLBW infants and infant deaths in ICUs [49]. Systemic fungal infections occur in 20% of babies weighing less than 1000 g [47]. VLBW and preterm infants are at increased risk of mycosis with *Candida* infection associated with the use of medical devices, including central lines for parental nutrition, endotracheal intubation, and broad-spectrum use of antibiotics [21]. Preterm infants are often exposed to prolonged use of corticosteroids, which impacts their immune function by diminishing T-cell activity and the alteration of glucose metabolism, while fungal species consume essential nutrients required by the host [48]. *C. albicans* is part of the female genital microbiota and can result in vertical transmission to the newborn and early-onset infection with non-albicans species associated with horizontal nosocomial transmission and late-onset infections. Once infection has been established, fungal species can disseminate, leading to deep-tissue infections in organs, including the heart, brain, eye, liver, kidney, and/or bone, leading to end-organ damage [50]. *Candida* meningitis or encephalitis is often not detected in cerebrospinal fluid and can remain asymptomatic in patients, with *Candida* pneumonia very rare in patients [51]. Importantly, Candidiasis is associated with moderate-to-severe cerebral palsy and neurodevelopmental impairment at 18 months of age in surviving infants [48]. Indeed, neurodevelopmental impairment has been reported in ca. 57% of patients [50]. Fortunately, studies show a decreasing prevalence of invasive *Candida* infection in neonatal ICUs, due to decreased use of broad-spectrum antibiotics, adherence to sterilisation protocols for medical devices, and fluconazole prophylaxis [49]. While *Aspergillus* infectious disease in infants is rare, *Aspergillus* fungi are the most frequent causes of neonatal IFIs in premature infants, with high rates of mortality [11]. In infants, *Aspergillus* infection is associated with pulmonary colonisation via the inhalation of spores, invasive catheters, dermal structure damage from medical devices, and wounds [49]. *Aspergillus fumigatus* is the most common species present, followed by *A. flavus*, *A. niger*, and *A. terreus*, with mortality rates of ca. 50% in severely immunocompromised patients [52]. The studies by Mohammad et al. (2023) describe the transmission of *A. flavus* to four premature infants from contaminated incubators and conclude that such species can persist environmentally in ICUs for several years [53]. Blood culture remains the standard for fungal diagnosis in neonates, with a success rate of ca. 50% and a detection time of up to 38 h [54]. For example, of 150 neonatal ICU patients tested of which 19 had culture-positive *Candida* meningoencephalitis, only 37% tested positive via blood culture [55]. The treatment of fungal infections in neonates is also challenging due to the high levels of MDR in fungal species and biocompatibility issues related to antifungal drug therapy. Voriconazole, for example, has a high rate of liver toxicity; achieving a suitable level of dosing is difficult in preterm infants [56]. Amphotericin B (AMPB), as monotherapy or combined with flucytosine, is administered for systemic fungal infections; AMPB, however, is associated with nephrotoxicity, hepatotoxicity, thrombocytopenia, and hypomagnesemia [20]. Fluconazole is better tolerated by infant patients and can be administered where susceptible strains are present as metaphylactic or prophylactic treatments [11]. 

### 2.3. Intestinal Dysbiosis in Neonatal Patients

The microbiota refers to the microbial species present on and in humans, which act in a commensal capacity. In terms of disease and the impact on health, the intestinal microbiota is considered extremely important. Studies describe the effect of an imbalance in this collection of microbial species termed dysbiosis and adult [57,58] and more recently infant health [59]. The intestinal microbiota consists of microbial species, which live in synergy with the host, while fungal, viral, and bacteriophage species are present, bacteria make up the majority of species present. Their essential role in the health and wellbeing of the host has been recognised and described elsewhere [21,57]. Evidence shows the role of the microbiota in metabolism, mucus production, the maturation and integrity of the intestinal barrier, and the development of innate and adaptive immune responses [58]. The interplay between the host immune system and colonising microbiota appears important in neonatal development [59]. Infant intestine colonisation is believed to begin in utero, where studies have detected microbial species in the meconium of infants containing *Staphylococcus*, *Enterobacteriaceae*, *Enterococcus*, *Lactobacillus*, and *Bifidobacterium species* [60]. The intestine of vaginally delivered full-term neonates is colonised by *Lactobacilli* and facultative anaerobes, including *Enterococcus*, *Enterobacteria*, *Streptococcus*, and *Staphylococcus*. These become replaced by species, including *Firmicutes*, *Bifidobacterium*, *Bacteroides*, and *Clostridia*, shortly after birth [59]. The mode of delivery and influence of breastfeeding on the composition of species present in neonates, factors which alter this microbiota balance include the exposure to antibiotics, caesarean section, and the exposure to pharmaceuticals, including anti-depressants, metformin, laxatives, and smoking [58]. Studies describe the effect of opioid exposure during pregnancy on neonatal intestine dysbiosis, with a loss of commensal beneficial species and the proliferation of pathogenic species [61]. Breastmilk increases the microbial diversity of the infant intestines and contains immune modulating factors, including IgA, mucin, lysozyme, anti-inflammatory molecules, growth factors, enzymes, oligosaccharides, polyunsaturated fatty acids, amino acids, and prebiotics, which are absent from milk formulas [62]. In preterm infants, Proteobacterial colonisation increases after birth, unlike full term infants. In the first 4 weeks after birth, low proportions of *Bifidobacteriaceae* and high proportions of *Enterobacteriaceae* and *Clostridiaceae* can result in dysbiosis [58]. Preterm infants display delayed colonisation of the intestines, with higher relative abundance of pathogens, such as *Klebsiella* species and *Clostridium* species [60]. Additionally, prolonged exposure to antibiotics is associated with intestinal dysbiosis in preterm infants, with significantly reduced beneficial *Bifidobacterium* and *Lactobacillus* present [63]. Studies demonstrate that VLBW infants with intestinal dysbiosis have increased risk of sepsis [63]. Furthermore, preterm infant dysbiosis is associated with the gastrointestinal disease NEC, with a mortality rate of ca. 30% [62]. Neurological and psychiatric issues, such as autism spectrum disorder, attention deficit hyperactivity disorder, schizophrenia, mental illnesses, and dementia, are also present in persons with intestinal dysbiosis [60]. At present, there are clinical trials ongoing, which aim to further investigate the relationship between the birth mode and gut microbiota on adverse health outcomes in infants over time, namely trials NCT03298334, NCT02567071, and NCT04173208 [64]. Currently, studies investigating the benefits of microbial transplants to correct dysbiosis, promote beneficial colonization, and prevent infections in neonates, are ongoing [65].

## 3. Advances in the Point-of-Care Detection and Treatment of Infectious Disease 

Early detection and treatment of infectious disease in neonates is essential to prevent mortality. Currently, blood culture, CSF, and urine are sampled in suspected cases of disease, including bacteraemia, meningitis, and pneumonia [2]. Microbiological culture, which is the gold standard, is time consuming and prone to false negatives, particularly in fungal diagnosis. Alarmingly, the detection via microbial culture ranges from 3–10% in early-onset sepsis and 16.7–33% in late-onset sepsis cases [66]. Additionally, clinical signs in neonates are nonspecific, where the immune system is not fully developed. Computed tomography (CT) may also be used to diagnosis fungal infection in patients. Diagnostic tests including the total white cell count, the absolute neutrophil count, the immature–total neutrophil ratio, have limited specificity and sensitivity [67]. The polymerase chain reaction (PCR) and 16S rRNA gene sequencing is a molecular methodology which can detect and identify microbial species in biological samples. Studies have shown excellent sensitivity and specificity of PCR assays when used for infectious disease [68]. PCR methods, however, require specialised equipment, are at risk of cross-contamination, and false positives, and are influenced by biological inhibitors. A paradigm shift away from the prophylactic use of broad-spectrum antimicrobials has occurred due to the emergence of MDR. Furthermore, increasing awareness on the impact of antibiotic use on neonatal intestinal dysbiosis and morbidity discourages such use of broad-spectrum antimicrobials. There is a lack of accurate and reliable diagnostic tools available for the early detection of infectious disease in infants, therefore, efficient triage and treatment remains challenging. The application of biomarkers, machine learning (ML), artificial intelligence (AI), and microfluidics technology may offer improved diagnostic methodologies. Point-of-care (POC) devices, such diagnostic methodologies, may provide fast, reliable, and accurate diagnostic aids for neonatal patients. 

### 3.1. Biomarkers of Inflammation

Infectious disease causes an immune response in the patient, triggering inflammation and the release of immune mediators. Such immune mediators include pro- and anti-inflammatory molecules, including the C-reactive protein (CRP), interleukins (IL), tumour necrosis factor α (TNF), and procalcitonin (PCT), which can be used as biological indicators of disease. These biomarkers can indicate physiological or pathological processes and the response to therapeutic interventions. Infectious disease biomarkers can be categorised as host-response biomarkers, for e.g., CRP, organ dysfunction biomarkers, and pathogen specific biomarkers, for e.g., antibody tests [69]. CRP and PCT have been used for the clinical diagnosis of inflammation, but do not differentiate the cause of inflammation and have relatively low sensitivity and specificity (Table 3) [21]. Both are evaluated in bacterial infections in infants, such as pneumonia and meningitis; however, their large and variable range hinders specificity. PCT rises rapidly in bacterial late- and early-onset sepsis and NEC and is not influenced by gestational age [70]. PCT, measured in cord blood, may be more beneficial as a discriminating marker of early-onset neonatal infection [71]. IL-6 and IL-8 also act as infectious disease biomarkers as their concentrations are elevated in infection; cutoff points, however, need to be established [34]. Notably, serum IL-6 in children is higher with Gram-negative BSIs than in Gram-positive BSIs, which may aid diagnosis protocols. The serum levels of these chemokines increase early in infant infection, but have short half-lives, limiting the detection window and require advanced measurement techniques [70]. Studies describe the potential of IL-6 and CRP, combined with improved sensitivity, for detecting neonatal sepsis [67]. The biomarker presepsin (P-SEP) has shown efficacy in detecting infectious disease in adults; studies have shown P-SEP may also aid in sepsis diagnosis in neonates. P-SEP is impacted by immature kidney function in preterm infants, which may limit its sensitivity; detection is improved, however, when combined with IL-6 and CRP [72]. Lactate dehydrogenase (LDH) in neonatal blood plasma can act as a biomarker, offering clinical benefits for several respiratory and metabolic disorders in infants, for e.g., NEC and asphyxia [73]. Biomarkers for the diagnosis of IFIs have been implemented clinically, namely the (1,3)-β-D glucan test (G test) and the galactomannan test (GM test) [74]. GM is an antigen produced by *Aspergillus* species, which can be detected in serum, plasma, CSF, and bronchoalveolar lavage (BAL), using the Platelia GM ELISA™ assay, approved by the USFDA [75]. The sensitivity and specificity values, however, vary from 30% to 90%, due to the small sample sizes and the presence of co-morbidities in patient cohorts [74]. Studies demonstrate the importance of repeat GM monitoring in infant patients to improve detection sensitivity [76]. T2Candida, Fungitell, Platelia Candida Antigen (Ag) Plus, and Platelia Candida Antibody (Ab) Plus assays are approved for diagnosis of candidemia in adults, but sensitivity in terms of clinical application remains an issue [77]. Studies demonstrate the efficacy of T2Candida to detect *Candida* in infant blood samples at the species level, providing accurate information on *Candida* BSIs in infants [78]. The findings, however, are influenced by small sample sizes, the absence of specific cutoff values, and the variability of infant immune responses. Identifying specific biomarker cutoff values in neonatal disease, which align with clinical symptoms, will improve patient triage and therapy and reduce antimicrobial use.

### 3.2. Machine Learning, Artificial Intelligence, and Microfluidics

Machine learning (ML), using mathematical algorithms and artificial intelligence (AI) to recognise, analyse, predict, and categorise medical data to diagnose disease states is showing promise in healthcare. The application of ML to analyse electronic health records (EHRs) to identify early signs of disease and to diagnose cancer has proven effective [81]. Studies demonstrate the success of ML in diagnosing sepsis and reducing sepsis mortality by ca. 12% [82]. The research by Robi and Sitote (2023) developed and applied a ML stacking model to categorise neonatal disease, including sepsis, birth asphyxia, NEC, and respiratory distress syndrome, which had improved efficiency compared to three other ML models, namely XGBoost (XGB), Random Forest (RF), and Support Vector Machine (SVM) [83]. ML has successfully predicted the likelihood of acquiring nosocomial *Clostridium difficile* infection and septic shock and to select suitable antibiotic therapy in adult patients [81]. The availability of clinical data, however, may hinder ML and AI progress in disease diagnosis, as algorithms require certain amounts of data for analysis. Obtaining high levels of data is often difficult due to missing values, poor record keeping, interpreter bias in terms of which variables are important, and variables such as the mode of transmission of infectious disease leading to poor predictive modelling [84]. Additionally, the variability of such data amongst infants of different ages, for e.g., full term vs. preterm infants, must be established. Gestational age can impact immune responses and the associated biomarkers. The use of Omics technology, which involves the application of high-throughput biochemical assays, based on cytomics, genomics, transcriptomics, proteomics, epigenomics, and metabolomics, may provide key data for ML algorithms. Monitoring the responses to infectious disease at a cellular and molecular level in patient cohorts may aid in establishing datasets and biomarkers with improved specificity and sensitivity. The deep learning model Sepis Watch^TM^ developed by the Duke Institute for Health Innovation has demonstrated the ability to provide early diagnosis of sepsis in clinical settings [21]. Organ-on-chip or 3-D biomimetic microfluidic assays are in vitro methods that combine microfluidic and tissue engineering to formulate a synthetic organ, with the physical parameters representative of in vivo conditions. These assays offer excellent rapid analysis of biological systems at omics levels, with good sensitivity and specificity [79]. The microfluidic LabDisk technology, developed as part of the ASCMicroPlat project, is designed to investigate the pathophysiology of neonatal sepsis; where LabDisk, using varying biomarkers, demonstrated sensitivity and specificity in detecting infectious disease [85]. SeptiCyte RAPID is an FDA-approved molecular test designed to detect sepsis as distinct from non-infectious inflammation, which detected sepsis within 1 h on the first day of ICU admittance [86]. The studies by Wang et al. (2024) applied a portable, integrated microfluidics methodology, which rapidly extracts DNA from sampling swabs and provided the detection of group B Streptococcus in infant patients with 100% specificity and selectivity, with a low limit of detection [87]. Currently, intrapartum antibiotic prophylaxis is administered to high-risk pregnant women to prevent group B *Streptococcus* transmission, with maternal GBS vaccines under development [88]. Fungi-on-Chip assays have been designed, allowing for studies of pathogenic yeast and filamentous fungi, such as *C. albicans* and *A. fumigatus* (aspergillosis-on-chip) infections [79]. The application of electrochemical biosensors may also offer diagnostic opportunities in infectious disease, due to their high sensitivity, selectivity, accuracy, miniaturisation, and transportability [89]. A biosensor is a device that measures biological or chemical reactions present in disease and produces a corresponding signal which can be detected [90]. Biosensors can provide real-time surveillance for monitoring infectious disease and POC diagnostics. The application of POC allows for rapid diagnosis, with increased sensitivity and specificity at a lower cost and must be “ASSURED”, i.e., affordable, sensitive, specific, user friendly, rapid and robust, equipment free, and deliverable to the end user [91]. The application of microfluidics and biosensors contribute to the production of portable, miniaturized, low cost, and highly integrated POC devices for POC diagnostics of various infectious diseases [92]. 

## 4. Conclusions

Neonatal infectious disease is a significant cause of infant mortality, with a higher prevalence in developing countries. Importantly, surviving patients often display neurodevelopmental issues and chronic disabilities if detection and treatment does not occur rapidly. Preterm infants are at greater risk; for example, preterm and VLBW infants with invasive fungal infections have a significantly higher incidence of mortality and adverse neurodevelopmental outcomes compared to non-invasive infections. The increasing rate of antimicrobial resistance in clinically relevant bacterial and fungal species further proliferates the issue and hinders antimicrobial stewardship. Pathogens are increasingly displaying multi- and pan-drug resistance to last-resort antimicrobials, prompting the WHO to announce a fungal priority pathogen list, accompanying the bacterial priority pathogen list. Critically important pathogens, *Klebsiella* and *Pseudomonas*, and fungal species, *Candida* and *Aspergillus*, are displaying resistance to front-line treatment options. Additionally, the impact of dysbiosis on infant and childhood health has emerged as a risk of inappropriate antimicrobial use. The development and implementation of efficient, effective, and sensitive point-of-care testing in neonatal disease triage is vital to safeguard infants. At present, microbiological testing remails the gold standard, which has serious limitation in terms of its application, sensitivity, and specificity. Biological indicators of inflammation, including CRP, PCT, and interleukins, offer some indication of disease in patients, but are not specific enough to determine infection and sepsis in infant cohorts. The development of more specific and sensitive biomarkers is essential to provide timely treatment at the early stages of disease. Currently, an ideal biomarker for neonatal infectious disease has not been identified due to the complexity of the infant immune system, biomarker cutoff values, and issues with sample sizes. Diagnostic methods, including machine learning, microfluidics, and biosensors, show potential as rapid, easy-to-use methodologies aligned with the WHO ASSURED criteria. The machine learning model, Sepsis Watch^TM^, has demonstrated efficacy in detecting sepsis, with the microfluidic LabDisk technology detecting sepsis in infant patients. Studies are warranted to established optimal and detailed datasets to allow for improved machine learning algorithms, which can be used in conjunction with biomarkers and biosensors in clinical settings. 

## Figures and Tables

**Table 3:** Advantages and limitations of novel detection methods for neonatal infectious disease.

Method	Example	Advantages	Limitations
Biomarkers	C-reactive protein (CRP), procalcitonin (PCT)	Standard test levels of PCT for preterm neonates available	Heterogenous nature of the host immune response. Lack of standardised analytical methods. Elevated in non-infectious inflammatory conditions. No standard concentrations identified especially in neonates. Absence of specific cutoff values.
	Cytokine interleukin 6 (IL-6)	Increases earlier than PCT and CRP in infection cases	
	Toll-like receptor presepsin	Elevated in the early stages of sepsis and is specific for infectious disease, with levels increasing within 2 h	
	The (1,3)-β-D glucan test (G test) and galactomannan test (GM test) for IFIs [74]	Application in haematology, respiratory, and IUC cases [74]	Sensitivity and specificity values range from 30% to 90%.
Machine Learning	Machine learning model, Sepsis Watch^TM^	Algorithms have successfully predicted sepsis, reduced hospital stay durations, and decreased mortality	ML requires sufficient data for analysis. Currently there are no regulatory frameworks designed for machine learning systems. Absence of high-quality clinical trials [21]. The outcomes are difficult to validate using external data.
Microfluidics	LabDisk technology for pathophysiology of neonatal sepsis. Aspergillosis-on-chip SeptiCyte RAPID, an mRNA test for sepsis.	Analysis of biological systems at cellular, molecular, genetic, and proteomic levels [79]. Fast turnaround times, low reagent volumes required, high integration capability, and improved sensitivity and specificity [80].	

## Data Availability

Not applicable.
